# Menthone lowers H3K27ac levels to inhibit *Fusarium proliferatum* growth

**DOI:** 10.3389/fmicb.2025.1533918

**Published:** 2025-01-22

**Authors:** Li-Yan Zhang, Tian-Tian Li, Hong-Xin Liao, Jin-Rui Wen, Hong-Yan Nie, Fu-Rong Xu, Xiao-Yun Liu, Xian Dong

**Affiliations:** ^1^School of Chinese Materia Medica, Yunnan University of Chinese Medicine, Kunming, China; ^2^Hubei Engineering Research Center for Protection and Utilization of Special Biological Resources in the Hanjiang River Basin, College of Life Sciences, Jianghan University, Wuhan, China

**Keywords:** antifungal activity, ribosomes, histone deacetylases, root rot disease, *Panax notoginseng*

## Abstract

**Background:**

The antifungal properties of essential oils (EOs) and their active constituents have been well documented. Histone acetylation is pivotal in modulating gene expression and influences biological processes in living organisms.

**Results:**

This study demonstrated that menthone, the primary component of *Mentha haplocalyx* EO, exhibited notable antifungal activity against *Fusarium proliferatum* (EC50 = 6.099 mmol/L). The treatment significantly inhibited hyphal growth, reduced spore germination rates from 31.49 to 4.95%, decreased spore viability from 46.88 to 20.91%, and reduced spore production by a factor of 17.92 compared with the control group while simultaneously enhancing cell membrane permeability. However, the direct relationship between menthone and histone acetylation in inhibiting *F. proliferatum* remains nebulous. Our RNA sequencing (RNA-seq) analysis identified 7,332 differentially expressed genes (DEGs) between the control and menthone-treated groups, 3,442 upregulated and 3,880 downregulated, primarily enriched in pathways related to ribosome biogenesis and energy metabolism. Chromatin immunoprecipitation sequencing (ChIP-seq) analysis revealed that menthone inhibited the growth of *F. proliferatum* by decreasing H3K27ac levels and interfering with the transcription of energy metabolism-related genes. By integrating the RNA-seq data with the ChIP-seq results, we identified 110 DEGs associated with reduced H3K27ac modification primarily associated with ribosome biogenesis. Menthone affected the growth of *F. proliferatum* by reducing the expression of ribosome biogenesis-related genes (*FPRO_06392*, *FPRO_01260*, *FPRO_10795*, and *FPRO_01372*).

**Conclusion:**

This study elucidated the mechanism by which menthone inhibits *F. proliferatum* growth from a histone acetylation modification perspective, providing insights into its application as an antifungal agent to prevent root rot in *Panax notoginseng*.

## 1 Introduction

*Panax notoginseng* (burk) Chen is a perennial herbaceous plant belonging to the Araliaceae family and recognized for its therapeutic applications in cardiovascular and orthopedic diseases (Xu et al., 2019). This is attributable to their unique active compounds and high medicinal value. However, due to its specific habitat requirements and extended cultivation cycle, *P. notoginseng* is susceptible to various diseases, of which root rot is one of the most detrimental. This can lead to the cracking and decay of the primary medicinal parts and their roots, resulting in significant economic losses (Ning et al., 2021). Root rot disease in *P. notoginseng* is primarily caused by pathogenic fungi belonging to the genus *Fusarium*. These pathogens adhere to the surface of the root system via hyphae, establishing colonies on the roots, which subsequently invade the vascular system and cause root rot (Zheng et al., 2022). Our research group has identified that, alongside *Fusarium oxysporum* and *Fusarium solani*, another pathogen responsible for root rot in *P. notoginseng* is *Fusarium proliferatum*, which can infect its roots and facilitate disease progression (Ma et al., 2019).

*F. proliferatum* is a major pathogenic fungus with a broad host range capable of causing root rot in various crops, including peanuts, tomatoes, and rice (Sun et al., 2022; Ye et al., 2020; Wang et al., 2021). Additionally, *F. proliferatum* can secrete toxins that may pose health risks to humans and animals (Alshannaq and Yu, 2017). *F. proliferatum* is a necrotrophic fungus widely distributed in the soil, where it can inhabit plant tissues and persist for many years by producing large quantities of chlamydospores. Under favorable environmental conditions, the spores of *F. proliferatum* germinate and disperse through wind and rain, leading to disease outbreaks (Nguyen et al., 2016; Panth et al., 2020). Currently, the management of root rot caused by *F. proliferatum* relies heavily on chemical pesticides, which can mitigate fungal diseases to some extent; however, prolonged use raises concerns regarding pesticide residues and environmental pollution, thereby posing threats to human health (Panth et al., 2020; Zeng et al., 2021).

Essential oils (EOs) are complex mixtures of volatile plant-derived compounds. In addition to their notable antimicrobial properties, these oils offer several advantages, including easy biodegradability and the absence of residues, which complicate the development of resistance by pathogens. Consequently, EOs represent a significant source of novel plant-based antimicrobial agents (Alonso-Gato et al., 2021). *Mentha haplocalyx* is a notable medicinal plant that belongs to the Lamiaceae family and is widely distributed across China, Korea, and Japan (Tang et al., 2024). Its EOs exhibit potent antibacterial activity against various pathogenic microorganisms (Karpiński, 2020). Research has indicated that menthone, a primary component of *M. haplocalyx* EOs, can disrupt the cell membranes of pathogens, causing the leakage of cellular contents and inhibiting cellular respiration, ultimately disturbing the internal equilibrium of the cells and resulting in cell death (Kang et al., 2019). Furthermore, menthone is one of the main constituents responsible for biological activities, such as antibacterial effects against *Helicobacter pylori*, antifungal properties against *Fusarium sambucinum*, and antibiofilm activity against methicillin-resistant *Staphylococcus aureus* (Piasecki et al., 2023; Pérez-Vázquez et al., 2022; Zhao et al., 2023). However, data on the molecular mechanisms by which menthone inhibits *Fusarium* species remain relatively limited.

Epigenetic modifications, including histone acetylation and methylation, play pivotal roles in regulating chromatin activity and gene transcription (Berger, 2007). Specifically, H3 acetylation at lysine 27 is frequently associated with activation of gene transcription (Zhang et al., 2021). In this study, we investigated the inhibitory effects of menthone on *F. proliferatum* by measuring EC50 values, spore germination rates, spore viability, spore yield, and cell membrane permeability. Additionally, we employed RNA sequencing (RNA-seq) and chromatin immunoprecipitation (ChIP) sequencing (ChIP-seq) analyses to examine the overall changes in gene transcription and histone acetylation levels following menthone treatment in *F. proliferatum*, thereby exploring its antifungal mechanism from the perspective of histone acetylation.

Our results indicate that menthone can compromise the cell membrane integrity of *F. proliferatum* and modulate the expression of genes associated with metabolic pathways related to ribosome biogenesis by reducing histone acetylation levels within this fungal strain. This finding highlights the potential antifungal targets of menthone and provides theoretical support for the development of targeted biological pesticides.

## 2 Methods

### 2.1 Fungal strains and growth conditions

The fungal strains used in this study were isolated from the roots of *P. notoginseng* exhibiting root rot. After isolating and purifying the strains, they were placed on potato dextrose agar (PDA) medium and incubated at a temperature of 28°C for a duration of 7 days. As previously described (Nie et al., 2024), DNA was extracted from the target strain and sequenced. Sequencing results were submitted to GenBank. A comparative analysis using NCBI BLAST revealed that the sequence exhibited 100% similarity to *F. proliferatum* (MH712158.1). The GenBank accession number is OP430570.1.

### 2.2 Evaluation of antifungal efficacy

Menthone was procured from Shanghai Yuanye Biological Technology Co., Ltd. (CAS. 14073-97-3, purity ≥97%). The effect of menthone on inhibiting the mycelial growth of *F. proliferatum* was evaluated using the mycelial growth rate method (Zhou et al., 2017). In a sterile environment, menthone was dissolved in a suspension of 20/1,000 Dimethyl sulfoxide (DMSO) and 1/1,000 Tween-80 (2-DMSO-T) and filtered to obtain a sterile filtrate. The menthone solution was diluted to various concentrations using a two-fold dilution method. The prepared menthone was incorporated into the PDA culture medium to create menthone-containing plates. A PDA plate containing only the 2-DMSO-T suspension was used as the negative control. Fungal blocks with a diameter of 5 mm were inoculated onto the central area of PDA plates containing different concentrations of menthone (0 mM, 0.5 mM, 1 mM, 2 mM, 4 mM, 8 mM, and 16 mM). The plates were incubated at 28°C in an incubator at constant temperature for 4 days. Each treatment comprised five replicates. Colony diameters were measured, and the inhibition rate was calculated. A linear regression equation was established using the least squares method, resulting in a toxicity regression equation. The EC50 value was subsequently derived from this toxicity regression equation (Zou et al., 2021). The percentage of mycelial growth inhibition is determined using the following formula. Inhibition rate (%) = (C − B)/(C) × 100, where C is the diameter of the negative control group colony (mm) and B is the diameter of the treatment group colony (mm).

### 2.3 Evaluation of the germination rate and viability of *Fusarium proliferatum* spores

Spore viability was assessed using a fluorescent staining method involving acridine orange and propidium iodide (Manzo-Valencia et al., 2016). The experimental procedure for spore germination was modified from the method described by Tanapichatsakul et al. (2020). Menthone was prepared according to the procedure outlined in section 2.2. Subsequently, the solution was transferred to a sterile EP tube (5 mL) containing 2 mL 1/3 PDA liquid culture medium. The solutions were thoroughly mixed to achieve a menthone concentration of 1 × EC50. An appropriate amount of the spore suspension was then added, with 2-DMSO-T serving as a negative control. Each treatment was performed in triplicates. After incubating the mixture on a shaker at 28°C and 180 rpm in the dark for 24 h, The rate of spore germination is determined using the following formula:


$$\begin{array}{l} \mathrm{Spore germination rate}=\left(\mathrm{Number of germinated spores}\right)/\\ \left(\mathrm{Total number of pores}\right)\times 100 \end{array}$$

### 2.4 Assessment of spore production in *Fusarium proliferatum*

Spore production was assessed as previously described with modification (Zheng et al., 2014). Menthone was added to Bilay’s culture medium at a concentration of 1 × EC50. Subsequently, 2 mL of the spore suspension, at a concentration of 1 × 10^6^ spores/mL, was added to each treatment group. The control group was treated with an equivalent volume of 2-DMSO-T as a solvent control. All samples were incubated in a thermostatic shaker at 28°C and agitated at 180 rpm in darkness for 5 days to facilitate shaking culture. Following incubation, spores were filtered through four layers of sterile cellulose acetate membranes [Sigma-Aldrich (Shanghai) Trading, MO, United States]. The resulting filtrate was thoroughly mixed, and samples were collected for analysis. The number of spores was quantified under a microscope using a hemocytometer. Each treatment included three biological replicates.

### 2.5 Assessment of membrane permeability

The permeability of the mycelial membrane was evaluated in accordance with the previously described methodology (Liu et al., 2022). A total of 2 g of mycelium was added to a conical flask containing 40 mL of sterile water, followed by menthone to achieve a final concentration equivalent to 1 × EC50. The conductivity was measured at 0, 1, 2, 3, 4, 5, 6, 7, and 8 h and recorded as D1. Concurrently, the conductivity without mycelia for each treatment group was measured and denoted as D0. After boiling the mycelium for 10 min, their conductivity was measured again and recorded as D2. DMSO was used as a negative control, with three biological replicates per treatment group. The formula was employed to calculate the relative conductivity. Relative conductivity = (D1 − D0)/D2 × 100%.

### 2.6 Assessment of the extracellular protein content of *Fusarium proliferatum* mycelium

The treatment method for the mycelia was consistent with that described in section 2.5. Samples were collected at time points of 0, 6, 12, 24, 48, and 72 h and subsequently centrifuged at 8,000 rpm for 5 min. The concentration of soluble proteins in the supernatant was determined using the Coomassie Brilliant Blue G-250 staining method (Hatada et al., 2006).

### 2.7 Sample preparation and RNA-seq analysis

As previously described (Liu et al., 2022), the mycelia of the fungus were collected following treatment with menthone for 24 h, immediately frozen in liquid nitrogen, and stored at −80°C for subsequent experiments. Total RNA extraction was performed following the manufacturer’s guidelines using TRIzol reagent. The quality of the extracted total RNA was assessed with a NanoPhotometer spectrophotometer (IMPLEN, CA, United States), a Qubit 2.0 fluorometer (Life Technologies, CA, United States), and an Agilent 2100 bioanalyzer (Agilent Technologies, CA, United States). A cDNA library was constructed at Novogene Technology Co., Ltd. (Beijing, China) and RNA-seq sequencing was performed. After purifying the cDNA and constructing the library, it was sequenced on an Illumina Novaseq6000 platform. The sequencing data were analyzed using Illumina Casava software (version 1.8) to remove adapter sequences and low-quality reads. Subsequently, the filtered reads were aligned to the reference genome of *F. proliferatum* (NCBI Taxonomy ID. 1227346) using the Hisat2 (version 2.2.1) software with default parameters.

Gene expression levels were determined as the number of transcript fragments per kilobase of transcript per million fragments (FPKM). The DEseq2 (version 1.22.1) software was used to analyzed DEGs between the two groups, with a screening standard of |log2Fold Change| ≥1 and false discovery rate (FDR) <0.05 (Ling et al., 2024). Finally, the results of the Gene Ontology (GO) and Kyoto Encyclopedia of Genes and Genomes (KEGG) pathway enrichment analyses were visualized using the online platform available at https://cloud.metware.cn.

### 2.8 Histone extraction and western blot experiment

Histones were prepared according to the instructions provided in the Histone Extraction Kit (BBproExtra^®^). The proteins obtained were separated by 15% sodium dodecyl sulfate-polyacrylamide gel electrophoresis (SDS-PAGE) and subsequently transferred to a polyvinylidene fluoride (PVDF) membrane. The membrane was then incubated with specific antibodies for hybridization. After hybridization, the membrane was blocked with 4% BSA in PBST (PBS-0.1% Tween20) at room temperature for 2 h. The primary antibodies used were anti-H3K27ac (ABconal, A2771, dilution factor is 1:10,000), anti-H3K4me3 (ABconal, A22146, dilution factor is 1:10,000), anti-H3K9ac (ABconal, A21107, dilution factor is 1:1,000), and anti-H3 (ABconal, A2348, dilution factor is 1:10,000). The hybridized membrane was washed three times with PBST for 10 min each before incubation with the primary antibody in PBST containing 4% BSA for an additional 2 h. Subsequently, the membrane was washed six times with PBST for 10 min each. This was followed by incubation with the corresponding secondary antibody, goat anti-rabbit IgG (Abbkine, A23220, dilution factor is 1:1,000), for 2 h. Finally, excess secondary antibody was removed by washing the membrane six times with PBST prior to signal detection in a dark room. The intensity of the bands was quantitatively analyzed using ImageJ software (Perrella et al., 2024).

### 2.9 ChIP-seq

ChIP experiments were conducted as previously described (Zhou et al., 2023), with modifications. Initially, the hyphae of *F. proliferatum* were ground into powder using liquid nitrogen. The chromatin was extracted by cross-linking with 1% formaldehyde under vacuum for a duration of 10 min. Following the completion of the cross-linking process, 2.5 M glycine was added and subjected to vacuum for an additional 5 min to effectively terminate the cross-linking reaction. Genomic DNA was fragmented into approximately 500 bp fragments through physical sonication, and ChIP was performed using an anti-H3K27ac antibody (A2771). The sequencing library was constructed following the protocol provided by the Illumina TruSeq^®^ ChIP Sample Prep Set A. Subsequently, sequencing was conducted on the Illumina HiSeq-PE150 platform, facilitated by Bioacme Biotechnology Co., Ltd.

### 2.10 Real-time fluorescence quantitative PCR and ChIP-qPCR

Total RNA was extracted from the mycelia using TRIzol reagent. First-strand cDNA was synthesized from the total RNA using a reverse transcription kit (TaKaRa). Real-time fluorescence quantitative PCR (RT-qPCR) was conducted on the CFX Manager 3.1 system (Bio-Rad) employing the SYBR Premix ExTaq kit (TaKaRa). In the RT-qPCR and ChIP-qPCR experiments, QTUB or input (unimmunoprecipitated chromatin sample) served as normalization controls. The relative expression levels of genes were analyzed using the $2^{-\Delta \Delta C_{T}}$ method. The primers used for RT-qPCR and ChIP-qPCR are listed in Supplementary Tables S1, S2, respectively.

### 2.11 Statistical analysis

All experiments conducted in this study were performed in triplicate for each sample, and the data were analyzed using one-way ANOVA with SPSS software version 19.0. Duncan’s multiple range test was used to assess the significance of differences (*p* < 0.05). All data are presented as mean ± standard deviation. The figures were generated using Adobe Illustrator 2022 and GraphPad Prism 9 software.

## 3 Results

### 3.1 Effect of menthone on the growth of *Fusarium proliferatum*

#### 3.1.1 Effect of menthone on hyphal growth of *Fusarium proliferatum*

**Figure 1:**
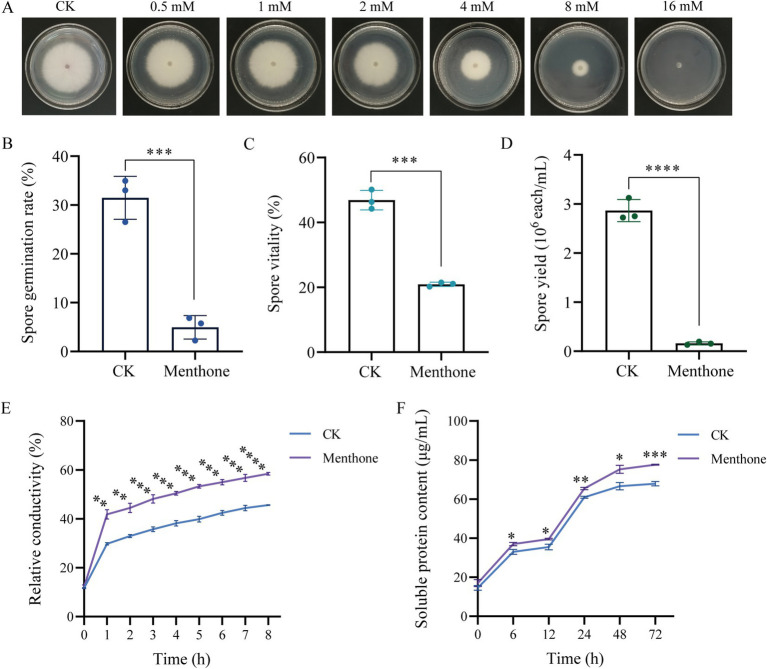
Antifungal effect of menthone against *F. proliferatum*. **(A)** Mycelial growth of *F. proliferatum* in PDA medium with various menthone concentrations. The effect of 1 × EC50 menthone on the spore germination rate **(B)**, spore vitality **(C)**, spore yield **(D)**, relative electrical conductivity **(E)**, and soluble protein content **(F)** of *F. proliferatum*. 2-DMSO-T (20/1,000 DMSO and 1/1,000 Tween-80 suspension) was used as a negative control (CK). ^*^p < 0.05, ^**^p < 0.01, ^***^p < 0.001, and ^****^p < 0.0001.

Menthone treatment significantly inhibited the hyphal growth of *F. proliferatum*. Under varying concentrations of menthone, a marked reduction in colony size was observed, with the inhibitory effect intensifying as the menthone concentration increased (Figure 1A). These findings suggest that menthone effectively suppresses hyphal growth. Additionally, the EC50 value of menthone against *F. proliferatum* was 6.099 mmol/L, indicating strong antifungal activity. Consequently, we selected 6.099 mmol/L (1 × EC50) as the menthone concentration for subsequent analyses.

#### 3.1.2 Effect of menthone on the germination and viability of *Fusarium proliferatum* spores

The results of the spore germination assay indicated that menthone significantly inhibited the germination of *F. proliferatum* spores (Figure 1B). At a treatment concentration of 1 × EC50, the spore germination rate of *F. proliferatum* was only 4.95%, which contrasted the control group rate of 31.49%. This inhibition factor was approximately 6.36 times lower than that observed in the control group. Furthermore, under identical treatment conditions, the viability of *F. proliferatum* spores decreased from 46.88 to 20.91% (Figure 1C). These findings suggest that menthone exerts a pronounced inhibitory effect on spore germination and viability of *F. proliferatum*.

#### 3.1.3 Effect of menthone on the sporulation of *Fusarium proliferatum*

The sporulation of *F. proliferatum* was evaluated microscopically by counting the number of spores in a filtered mycelial suspension using a hemocytometer. The sporulation rate for the control group was 2.867 × 10^6^ spores/mL. In contrast, the menthone-treated group demonstrated a significantly reduced sporulation rate of only 0.160 × 10^6^ spores/mL, indicating an approximate reduction factor of 17.92 compared with the control group (Figure 1D). These results suggest that menthone exerts inhibitory effects on normal growth and spore development of *F. proliferatum*, leading to substantial impairment of its reproductive capacity.

#### 3.1.4 Effect of menthone on the relative conductivity of *Fusarium proliferatum*

The cell membrane plays a crucial role in preserving the structural integrity of the cell. When the cell membrane is compromised, normal physiological metabolism becomes impaired, and the leakage of cellular contents results in an increased conductance of the extracellular fluid. The effect of menthone on the membrane permeability of *F. proliferatum* was assessed by measuring its relative conductivity. Compared with the control group, a significant increase in the relative conductivity of *F. proliferatum* hyphae was observed with prolonged menthone treatment (Figure 1E). These findings indicate that menthone disrupts the membrane structure of *F. proliferatum*, resulting in electrolyte leakage and elevated extracellular conductivity.

#### 3.1.5 Effect of menthone on the soluble protein content of *Fusarium proliferatum*

The soluble protein content of *F. proliferatum* mycelia is shown in Figure 1F. Following menthone treatment, soluble protein content in the extracellular space of *F. proliferatum* mycelia gradually increased. Notably, protein levels exhibited a significant increase from 12 to 24 h, peaking at 72 h before stabilizing. Throughout the period from 6 to 72 h, the concentration of soluble protein in the treatment group was consistently and significantly higher than that observed in the control group. These findings suggest that menthone disrupts the cell membrane integrity of fungal cells, resulting in altered cell permeability and the subsequent leakage of cellular contents, inhibiting normal fungal growth.

### 3.2 Transcriptomic analysis of *Fusarium proliferatum* following menthone treatment

**Figure 2:**
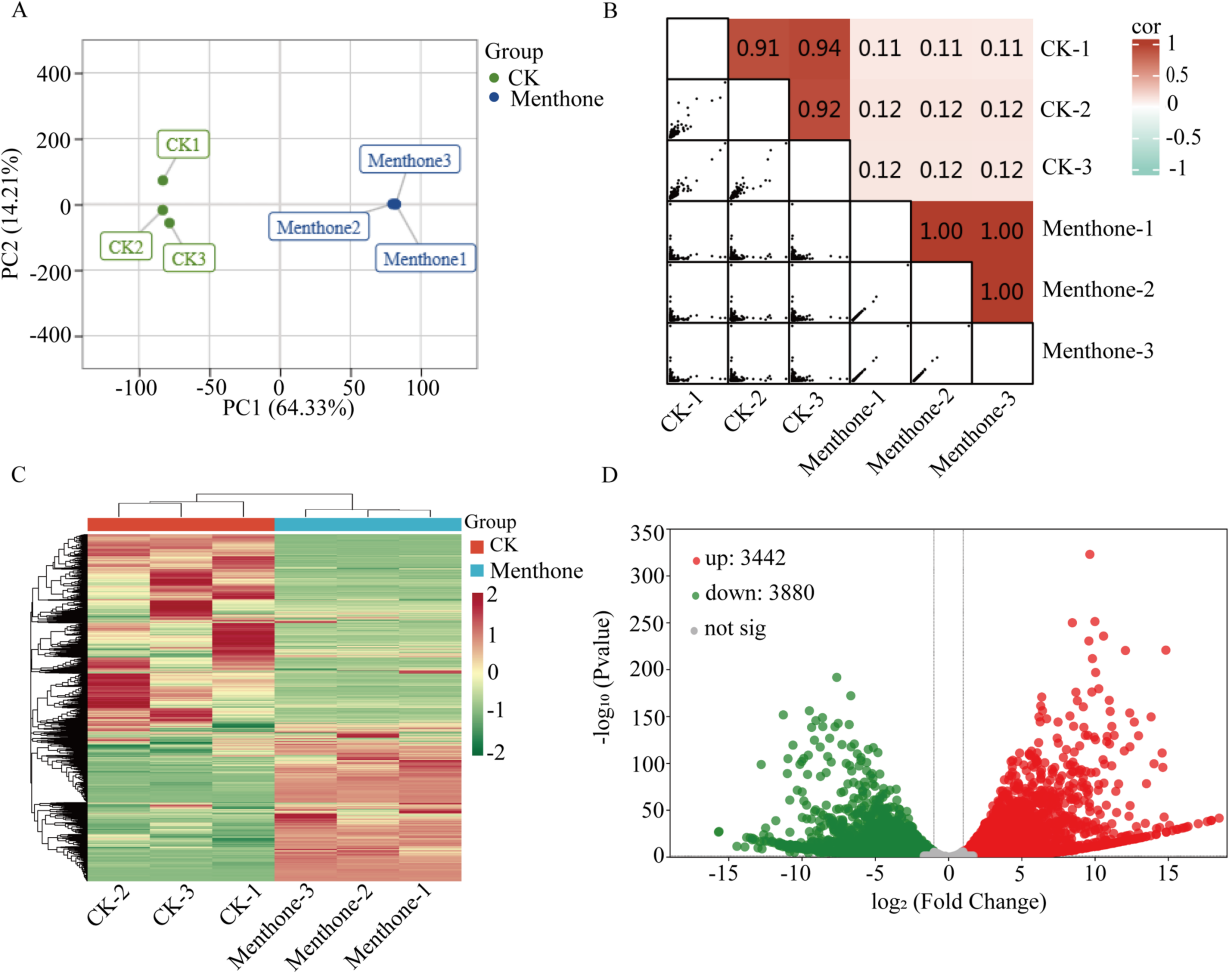
Preliminary analysis of transcriptomic data. **(A)** Principal component analysis score plot for all samples. The two treatments were control (CK) and menthol. **(B)** Heatmap of gene expression correlation. The color indicates the level of the correlation coefficient, and the number represents the correlation coefficient. **(C)** Clustering heat map of DEGs. **(D)** Volcano map of DEGs. The green and red colors represent downregulation and upregulation, respectively.

To elucidate the antifungal mechanism of menthone against *F. proliferatum*, we performed transcriptome sequencing analysis of samples treated with menthone using the Illumina NovaSeq 6000 platform. After data cleaning and quality control, high-quality reads were generated for each independent sample, ranging from 21.25 to 31.77 million reads. These high-quality reads were successfully mapped to the reference genome of *F. proliferatum*, achieving mapping rates exceeding 90%, indicating the high level of reliability of our transcriptomic data (Supplementary Table S3). Principal component analysis (PCA) and correlation analyses demonstrated good biological repeatability within the sample groups, confirming the reliability of our results (Figures 2A,B).

**Figure 3:**
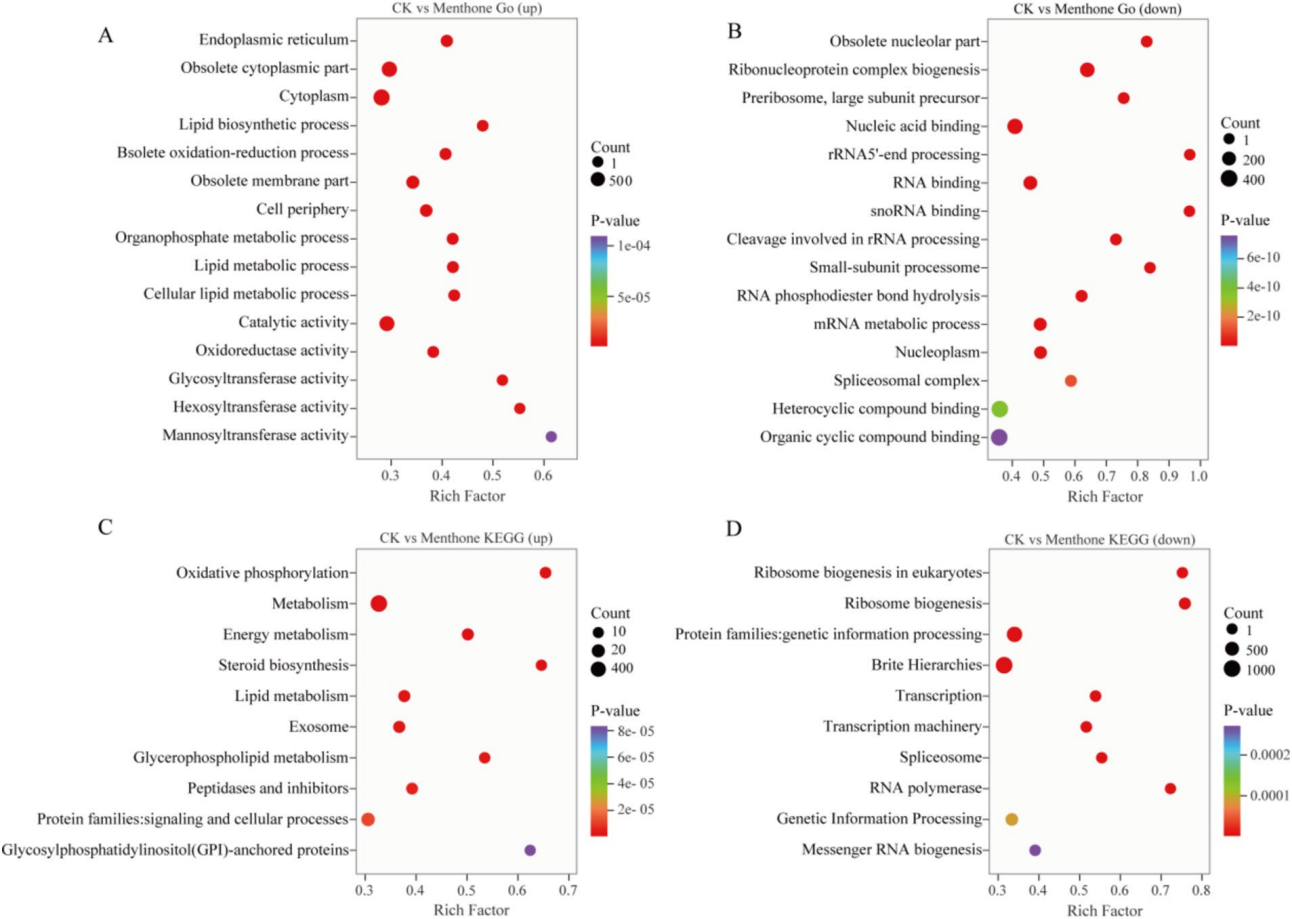
Gene Ontology (GO) and Kyoto Encyclopedia of Genes and Genomes (KEGG) enrichment analysis of differentially expressed genes (DEGs). GO enrichment analysis of upregulated **(A)** and downregulated **(B)** DEGs between CK and menthone. KEGG enrichment analysis of upregulated **(C)** and downregulated **(D)** DEGs between CK and menthone. The *x*-axis represents the enrichment factor, while the *y*-axis indicates the pathway names. The color of the points reflects the *p*-value, with red indicating more significant enrichment and the size of the points corresponds to the number of DEGs.

To further explore gene expression patterns, we identified DEGs using criteria of |log2Fold Change| ≥1 and false discovery rate (FDR) <0.05 for screening purposes. Our analysis revealed 3,442 upregulated genes and 3,880 downregulated genes in the menthone-treated group compared with the control group (Figures 2C,D). These findings suggest that menthone modulates the growth and development of *F. proliferatum* by inhibiting the expression of specific genes. Subsequently, the DEGs following menthone treatment were subjected to Gene Ontology (GO) functional enrichment analyses. The results indicated that upregulated DEGs were predominantly enriched in pathways related to the endoplasmic reticulum, cytoplasm, and lipid biosynthetic and metabolic processes. In contrast, downregulated DEGs were significantly enriched in pathways associated with ribonucleoprotein complex biosynthesis and nucleic acid-binding activities, including RNA- and snoRNA-binding pathways (Figures 3A,B).

To further investigate the biological functions of these DEGs in the growth and development of *F. proliferatum*, we conducted a Kyoto Encyclopedia of Genes and Genomes (KEGG) pathway enrichment analysis of the DEGs, selecting the top 10 most significantly enriched pathways (Figures 3C,D).

DEGs upregulated by menthone treatment were predominantly enriched in oxidative phosphorylation, energy metabolism, steroid biosynthesis, and lipid metabolism. These findings suggest that menthone treatment enables *F. proliferatum* to mitigate membrane damage caused by menthone by enhancing steroid biosynthesis and lipid metabolism while simultaneously boosting oxidative phosphorylation and energy metabolism pathways to supply cellular energy. Conversely, the downregulated DEGs were primarily enriched in ribosome biogenesis, ribosome biogenesis in eukaryotes, transcription, and spliceosomes. The regulation of ribosomal biogenesis is a critical signal for cell growth and proliferation (Li et al., 2021). We hypothesized that menthone influences ribosome biogenesis in eukaryotes and other metabolic processes, affecting fungal protein synthesis and inhibiting the nutritional growth of *F. proliferatum*.

### 3.3 Analysis of histone modifications in *Fusarium proliferatum* following menthone treatment

**Figure 4:**
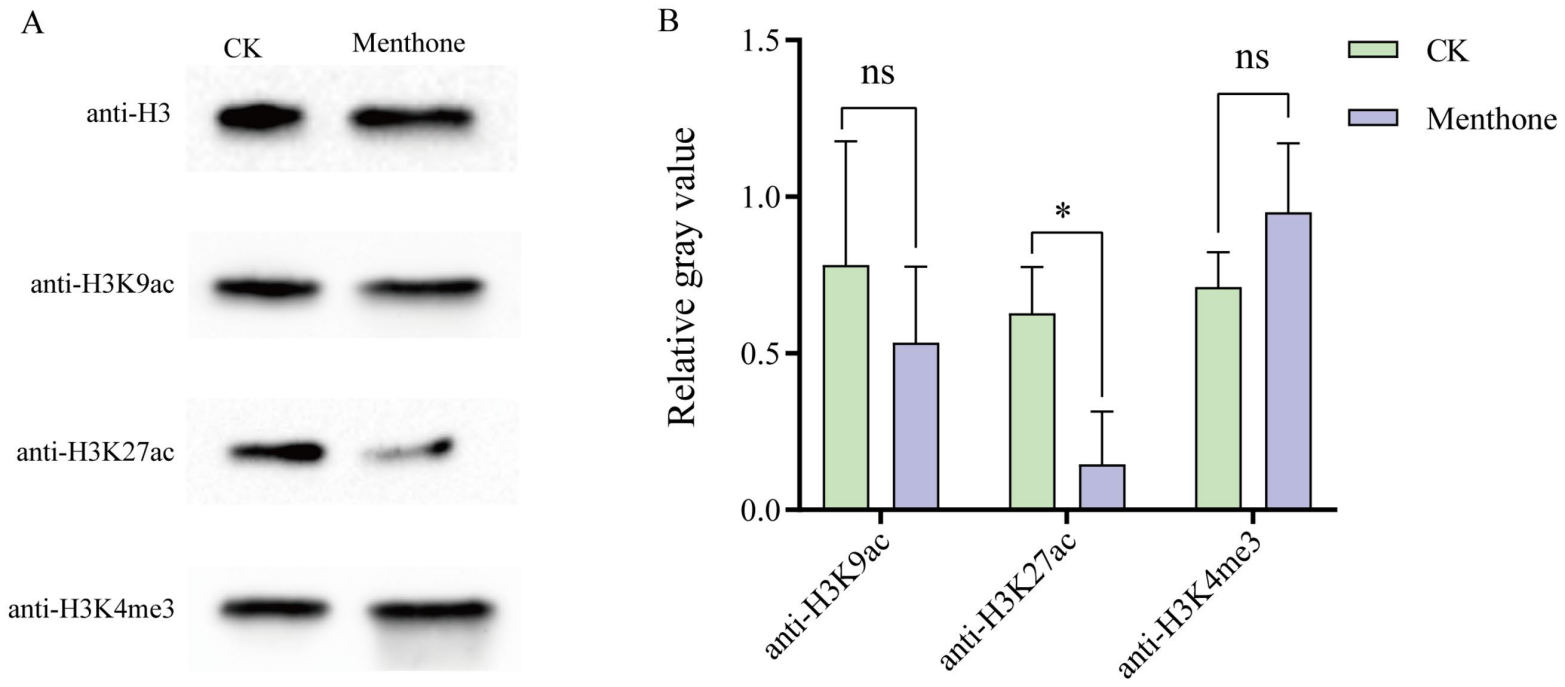
Menthone modulates the level of histone acetylation modification in *F. proliferatum*. **(A)** The levels of H3K9ac, H3K27ac, and H3K4me3 in *F. proliferatum* were detected using western blotting. Anti-H3 was used as the loading control. **(B)** Changes in relative gray values for the levels of histone acetylation and methylation modification in different treatment groups. ^*^p < 0.05, ns, not significant.

To elucidate the antifungal mechanism of menthone, we assessed the changes in histone modification levels using western blotting following menthone treatment. The results indicated that compared with the control group, the acetylation level of H3K27 was significantly reduced in *F. proliferatum* post-menthone treatment. No significant alterations were observed at other lysine sites of histone H3 (Figure 4; Supplementary Figure S1). This suggests that menthone primarily modulates the histone acetylation of H3K27 in *F. proliferatum*.

Previous studies have established a close relationship between histone acetylation and gene activation. In this study, H3K27ac levels decreased following menthone treatment, which might have inhibited related gene expression. Therefore, we hypothesized that menthone inhibits the growth of *F. proliferatum* by suppressing histone acetylation.

### 3.4 ChIP-seq analysis of *Fusarium proliferatum* following menthone treatment

**Figure 5:**
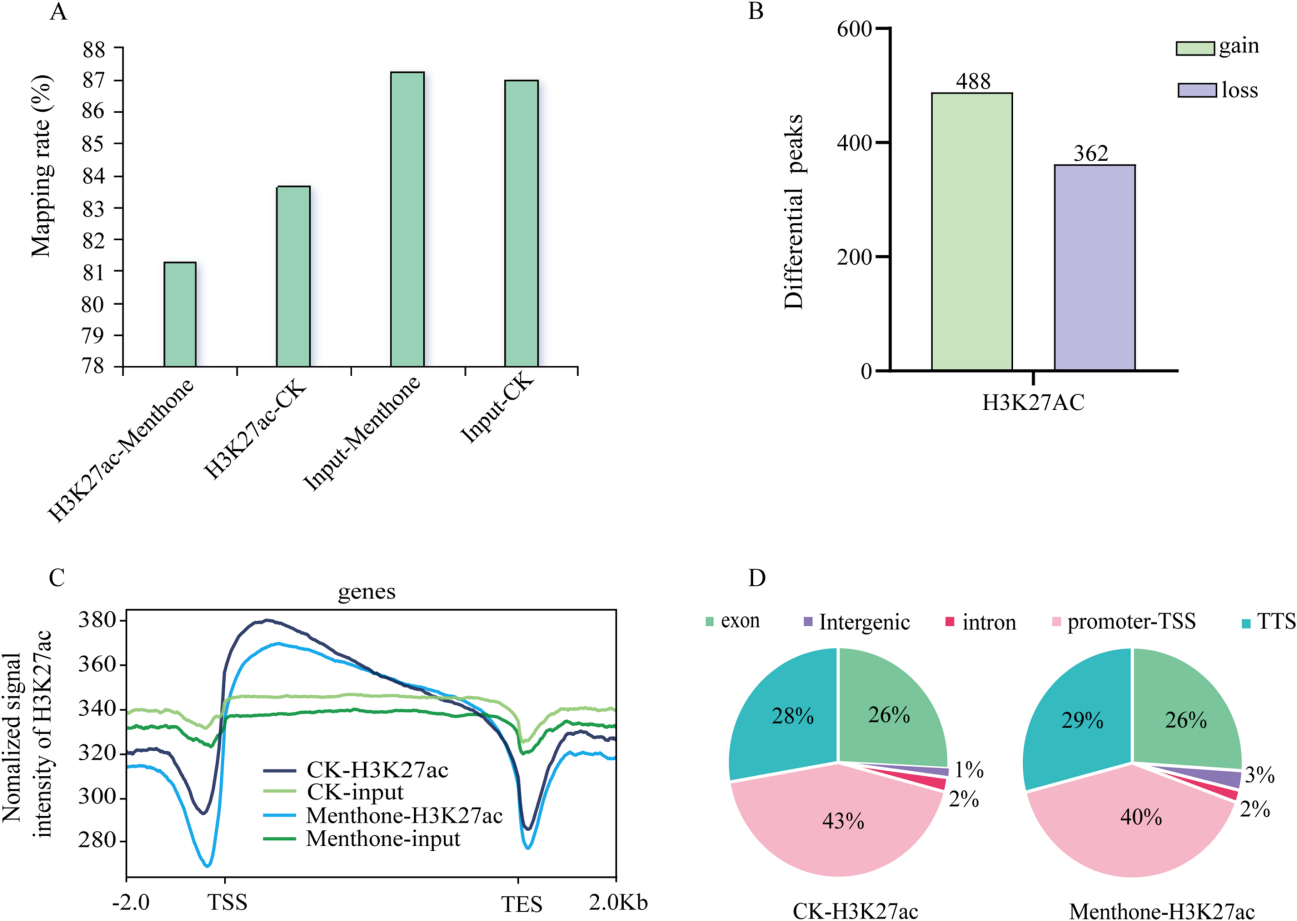
Preliminary analysis of ChIP-seq data. **(A)** Changes in H3K27ac levels in the menthone treatment group compared with the control group. **(B)** The number of genes associated with peaks that exhibit differences between the menthone treatment group and the control group. In comparison to the control group, genes within the menthone treatment group were classified as gain or loss genes based on the presence or absence of H3K27ac peaks. **(C)** Distribution of H3K27ac peaks at TSS-gene-TES across different treatment groups. TSS, transcriptional start site; TES, transcriptional end site. **(D)** The distribution of H3K27ac across various gene elements in different treatment groups.

To investigate the dynamic changes in H3K27 acetylation in *F. proliferatum*, we performed a ChIP-seq analysis. The results indicated that the number of genes enriched in high and low acetylation modifications affected by H3K27ac following menthone treatment was 488 and 362, respectively, when compared to the control (Figure 5B). The mapping rates for both ChIP-seq samples exceeded 80%, with approximately 40% of the modified binding sites located near the transcription start site (Figures 5A,D and Supplementary Table S4). Furthermore, H3K27 acetylation peaks were significantly enriched at transcription start sites across different treatment groups (Figure 5C). This finding aligns with the previously reported distribution patterns of histone markers (Papait et al., 2013), validating the authenticity of our sequencing data.

### 3.5 Menthone treatment reduces H3K27ac to inhibit the ribosome biogenesis of *Fusarium proliferatum*

**Figure 6:**
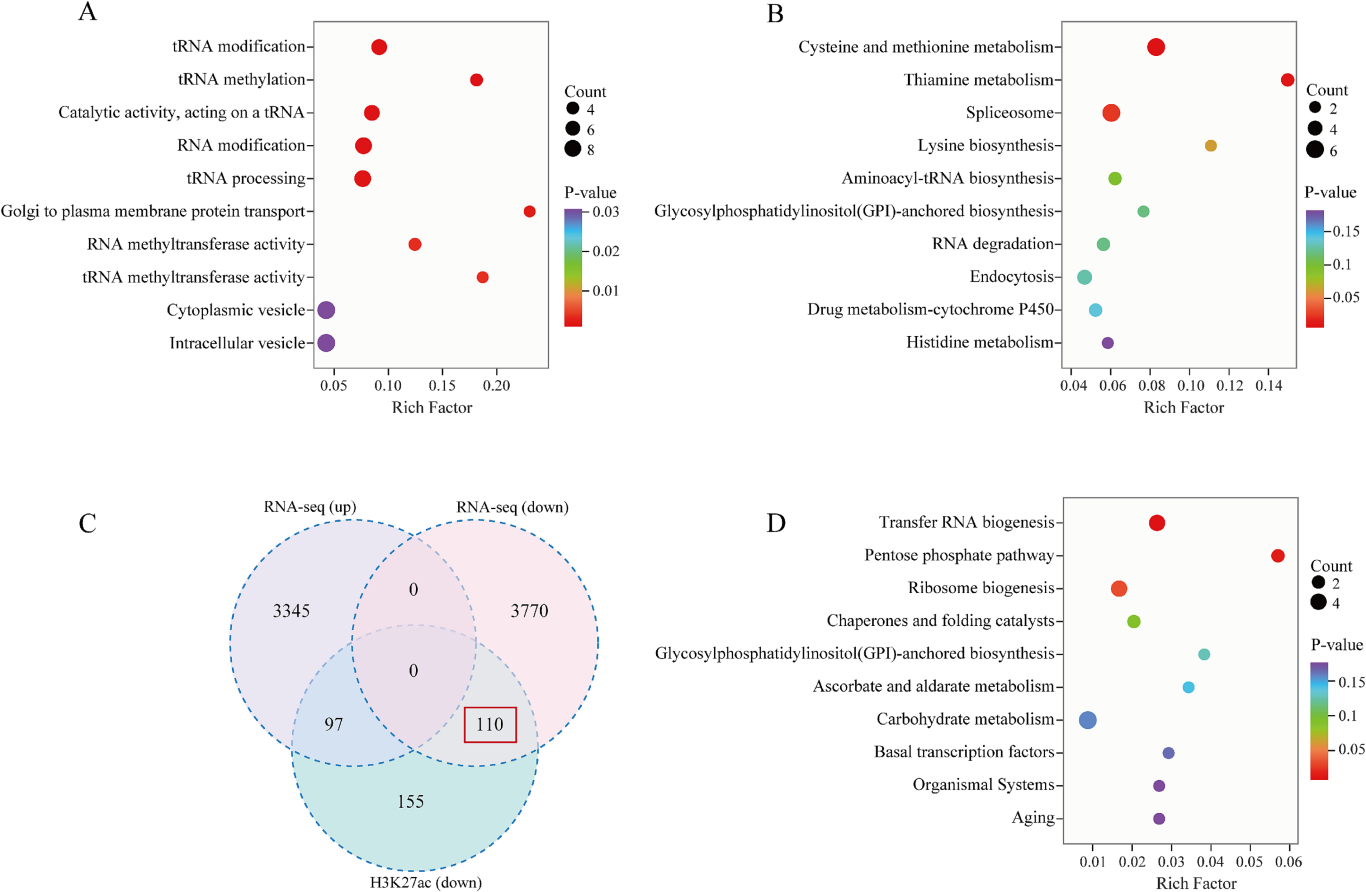
Analysis of genes associated with differential H3K27ac peaks following menthone treatment in *F. proliferatum*. **(A)** Gene Ontology (GO) enrichment analysis of low acetylation genes in the menthone treatment group compared with the control group. **(B)** Kyoto Encyclopedia of Genes and Genomes (KEGG) pathway enrichment analysis of low acetylation genes in the menthone treatment group versus the control group. **(C)** Venn diagram illustrating low acetylation genes alongside transcriptionally upregulated and downregulated genes in the menthone treatment group relative to the control group. **(D)** KEGG enrichment analysis of low acetylation and transcriptionally downregulated genes (*n* = 110) within the menthone treatment cohort.

Combining the results from the western blot analysis, we focused on the regulation of genes downregulated by histone modifications. Initially, we conducted GO enrichment analysis of the differentially acetylated peak genes to investigate the biological functions associated with these modified genes. Our findings revealed that these genes were predominantly enriched in tRNA modification, methylation, and processing (Figure 6A). Furthermore, KEGG enrichment analysis indicated that these genes were significantly enriched in cysteine and methionine metabolism and the spliceosome pathway (Figure 6B). These results imply that menthone treatment influences the functionality of the *F. proliferatum* spliceosome, resulting in disrupted gene expression, ultimately exerting antifungal effects and affecting the normal life processes of the fungus.

To investigate the regulatory mechanism of H3K27ac on gene transcription in *F. proliferatum*, we conducted a combined analysis of RNA-seq and ChIP-seq data to identify the transcripts associated with H3K27ac peaks following menthone treatment. Our comparative analysis revealed 362 low-acetylation peaks in the menthone-treated group, corresponding to 207 transcripts. Notably, the expression levels of 110 genes were significantly downregulated (Figure 6C). This finding suggests that after menthone treatment, H3K27ac primarily influences the downregulation of *F. proliferatum* genes, thereby inhibiting their growth and development. KEGG pathway enrichment analysis was performed to elucidate the pathways involved in the co-downregulated genes (*n* = 110) with low H3K27ac acetylation, indicating a significant enrichment within the ribosome biogenesis pathway (Figure 6D and Supplementary Tables S5, S6).

### 3.6 RT-qPCR and ChIP-qPCR for validation

**Figure 7:**
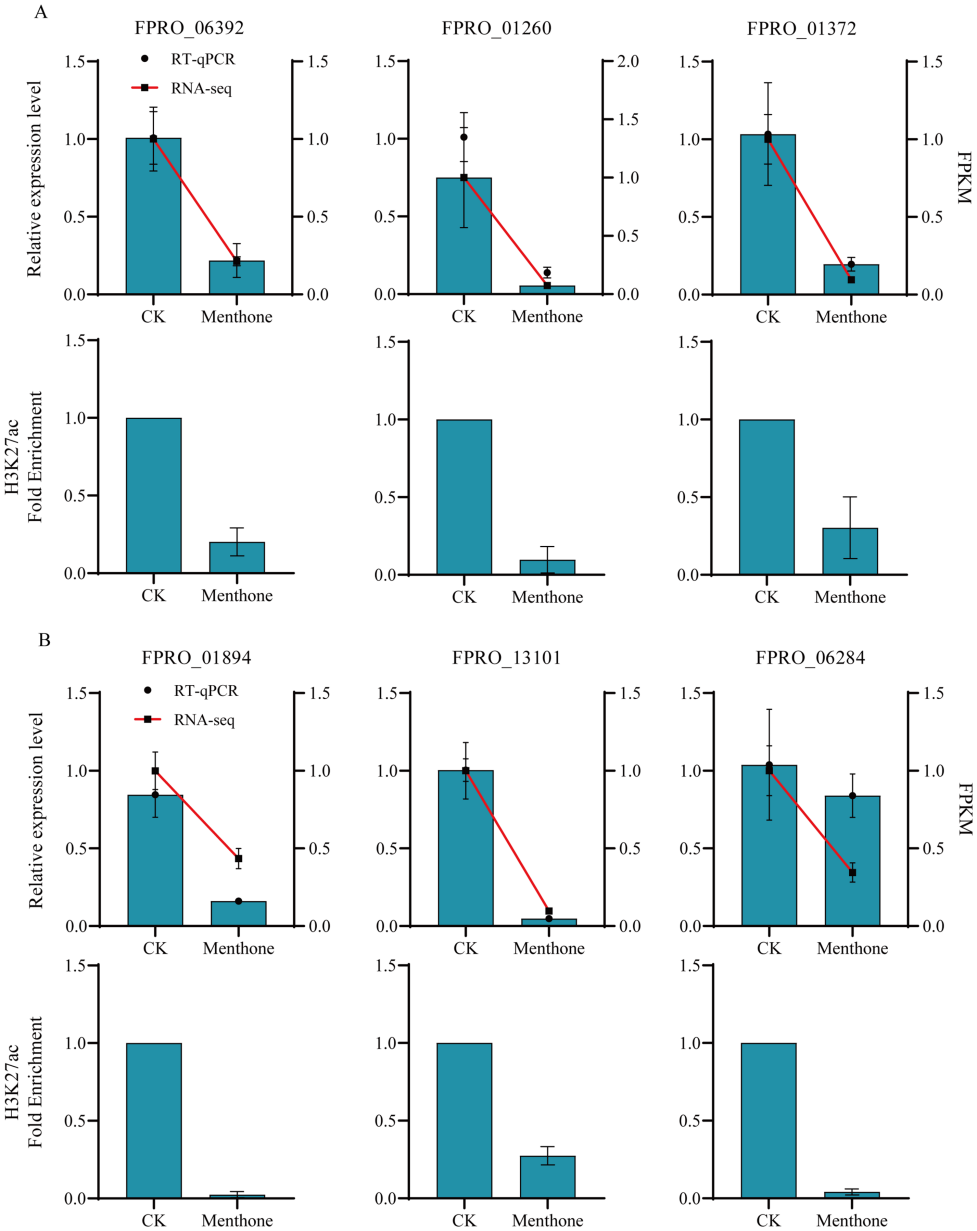
Validation of differential gene expression in *F. proliferatum* following menthol treatment assessed using RNA-seq and H3K27ac analysis. The RT-qPCR and ChIP-qPCR validation of ribosome biogenesis-related genes **(A)** and spliceosome and tRNA biosynthesis-related genes **(B)** in *F. proliferatum* following menthol treatment are presented.

Several relevant genes were selected for RT-qPCR and ChIP-qPCR to validate the accuracy of the transcriptome and ChIP-seq data. The results indicated (Figure 7) that treatment with menthone significantly downregulated the expression levels of genes involved in ribosome biogenesis (*FPRO_06392*, *FPRO_01260*, and *FPRO_01372*), spliceosome-related genes (*FPRO_01894* and *FPRO_13101*), and tRNA biosynthesis-related genes (*FPRO_06284*) in *F. proliferatum* compared to the control group. This finding is consistent with the transcriptome sequencing results, suggesting that the transcriptomic data are reliable.

Furthermore, after menthone treatment, there was a significant decrease in the H3K27ac modification levels of these genes relative to those in the control group. Consequently, reducing H3K27ac levels in the promoter regions of the relevant genes in *F. proliferatum* inhibited transcriptional activation. Our findings demonstrate that menthone treatment leads to decreased acetylation modifications at H3K27, suppressing gene transcription in *F. proliferatum*, subsequently affecting its growth and development.

## 4 Discussion

Prolonged use of chemical pesticides poses a significant threat to human health and the environment, necessitating safe and effective new natural antimicrobial agents (Acheuk et al., 2022). Research has demonstrated that the essential oils (EOs) derived from *M. haplocalyx* exhibit a broad spectrum of antimicrobial properties against various pathogenic microorganisms, including *F. oxysporum*, *F. proliferatum*, and *Rhizopus stolonifera* (Soliman et al., 2022; Kumar et al., 2016; Yan et al., 2021). Menthone, which constitutes approximately 27.39% of the total content in *M. haplocalyx* EO, has strong inhibitory effects on pathogenic fungi such as *Fusarium verticillioides*, *Aspergillus niger*, and *Botrytis cinerea* (Felšöciová et al., 2020; Dambolena et al., 2008; Camele et al., 2021). In this study, we conducted *in vitro* antifungal experiments to demonstrate that menthone significantly inhibited hyphal growth, spore germination, spore viability, and yield of *F. proliferatum* (Figure 1). The cell membrane plays a crucial role in the maintenance of cellular integrity. When compromised, normal physiological activities are hindered, leading to the leakage of cellular contents (Andrade-Ochoa et al., 2021). In this study, menthone disrupted the integrity of the cell membrane in *F. proliferatum* while increasing its permeability (Figure 1), as evidenced by the elevated extracellular relative conductivity and soluble protein levels. These findings demonstrate the inhibitory function of menthone against the growth and development of *F. proliferatum*, which holds promise as a potential antifungal agent for controlling root rot associated with *P. notoginseng*.

Transcriptome sequencing has been used to elucidate the molecular mechanisms by which various organisms respond to stress at the molecular level. This approach provides insights into the DEGs involved in specific biological processes (Shi et al., 2023). In the present study, following menthone treatment, we identified 7,332 DEGs in *F. proliferatum* (Figures 2C,D). KEGG enrichment analysis revealed that these DEGs were significantly associated with oxidative phosphorylation, energy metabolism, lipid metabolism, ribosome biogenesis, and ribosome biogenesis in eukaryotic organisms, with a notable degree of enrichment (Figure 3). These findings indicate that menthone alters gene expression in *F. graminearum*, with most DEGs participating in fundamental physiological processes within the organism. In our previous study, we demonstrated that β-caryophyllene oxide, a constituent of *M. haplocalyx* EO, effectively reduced histone acetylation levels in *F. proliferatum*. This reduction subsequently inhibited ribosome biosynthesis and function, ultimately impairing the normal growth and pathogenicity of this pathogen (Liao et al., 2024).

Histone acetylation is crucial in epigenetic regulation that significantly influences various cellular processes, such as cell morphology, metabolic pathways, and protein synthesis (Yao et al., 2023; Wang et al., 2024). Dynamic regulation of histone acetylation primarily involves histone acetyltransferases (HATs) and histone deacetylases (HDACs), HATs facilitate the transfer of an acetyl group from acetyl-CoA to lysine residues on histones, whereas HDACs modulate gene expression by removing these acetyl groups from histones (Zhao et al., 2023). By modulating histone acetylation levels, HDACs can influence gene expression in critical biological processes. In the present study, menthone treatment reduced the level of H3K27 acetylation in *F. oxysporum* (Figure 4), inhibiting the expression of associated genes and adversely affecting normal growth. Typically, a decrease in histone acetylation correlates with increased HDAC activity. This shift results in a more compact chromatin structure, which inhibits gene transcription (Li et al., 2023). Previous studies have demonstrated that HDAC plays a vital role in the growth, development, and pathogenesis of plant pathogenic fungi (Brosch et al., 2008). In *Magnaporthe oryzae*, the histone deacetylase MoRpd3 plays a crucial regulatory role in fungal growth and pathogenicity. MoRpd3 overexpression in *M. oryzae* results in reduced hyphal production and loss of pathogenicity (Lin et al., 2021). This study showed that menthone promoted the expression of genes encoding the histone deacetylases *FPRO_03415* and *FPRO_13046* (Supplementary Table S7). Therefore, menthone can enhance the expression of these genes, thereby influencing histone acetylation and inhibiting the growth of *F. proliferatum*.

These findings suggest that menthone functions as an epigenetic modulator with the potential to inhibit fungal growth, potentially facilitating future research on HDAC activators. As documented in the existing literature, HDACs have been shown to modulate the acetylation of ribosomal proteins, thereby influencing the process of ribosome assembly (Xu et al., 2021). Furthermore, it has been demonstrated that the repression of genes involved in ribosome biogenesis contributes to the inhibition of mycelial growth and reduces the pathogenicity of the fungus *Fusarium* (Li et al., 2021). To elucidate the mechanism by which H3K27ac regulates gene transcription, we performed a combined analysis of RNA-seq and ChIP-seq data. This analysis revealed that 110 downregulated DEGs by low levels of H3K27ac modification (Figure 6C). Further examination indicated that most genes associated with ribosome biogenesis were downregulated, including *FPRO_06392*, *FPRO_01260*, *FPRO_10795*, and *FPRO_01372*, which encode heat shock proteins BUD20, NOC4, POP3, and HSP70, respectively. BUD20 plays a primary role in bud site selection. Research has demonstrated that this protein is involved in the epigenetic regulation of cell polarity during bipolar budding, and its reduced or absent expression leads to significant budding defects (Ni and Snyder, 2001). NOC4 belongs to the NOC family and collaborates with other NOC proteins in the processing and modification of preribosomal RNA, thereby playing a crucial role in ribosome biogenesis. The absence of NOC4 blocks ribosome synthesis, inhibiting cell growth (Milkereit et al., 2003). POP3 is a protein subunit of RNase MRP and RNase P primarily involved in pre-tRNA processing and is essential for cell viability. POP3 deletion results in cell death (Dichtl and Tollervey, 1997; Perederina et al., 2023). Ahamad et al. (2016) and Ahamad et al. (2021) demonstrated that in *Schizosaccharomyces pombe*, POP3 is critical for oxidative stress resistance; cells deficient in POP3 exhibit increased sensitivity to hydrogen peroxide and phenol red, adversely affecting spore formation and overall cell viability. HSP70 is a highly conserved molecular chaperone essential in fungal growth and development, secondary metabolite synthesis, and virulence. The deletion of HSP70 markedly affects hyphal growth and spore formation in *Clonostachys rosea* (Sun et al., 2019).

In summary, menthone regulates the expression of related genes by reducing the acetylation of H3K27ac in *F. proliferatum*, thereby influencing fungal growth. The decreased expression of *BUD20*, *NOC4*, *POP3*, and *HSP70* may be crucial factors contributing to the inhibition of *F. proliferatum* spore germination, cell viability, hyphal growth, and spore production. Therefore, investigating histone acetylation modifications will provide novel insights into the antifungal mechanisms of menthone against the pathogen responsible for root rot in *P. notoginseng*. This study provides a theoretical foundation for developing targeted biological pesticides.

## 5 Conclusion

Our study demonstrates that menthone significantly inhibits *F. proliferatum* by reducing mycelial growth, spore germination rates, viability, and yield while increasing cell membrane permeability. Transcriptome sequencing and ChIP-seq analyses revealed that menthone treatment reduced H3K27 acetylation levels in *F. proliferatum* and inhibited the expression of genes related to ribosome biogenesis pathways, ultimately suppressing growth. Our study highlights the potential antifungal targets of menthone through histone acetylation modification, provides a theoretical basis for its use as an antimicrobial agent against root rot disease caused by *P. notoginseng*, and offers insights into developing targeted biopesticides.

## Data Availability

The RNA-seq data and ChIP-seq data presented in the study are deposited in the NCBI repository, accession number PRJNA1209135 and SAMN46191626.
