# Supplementary material for: Menthone lowers H3K27ac levels to inhibit Fusarium proliferatum growth
Source: Front Microbiol. 2025 Jan 22;16:1533918. doi: 10.3389/fmicb.2025.1533918 (PMC11794811; doi:10.3389/fmicb.2025.1533918)
Supplement: Supplementary file 1 [file Data_Sheet_1.docx]

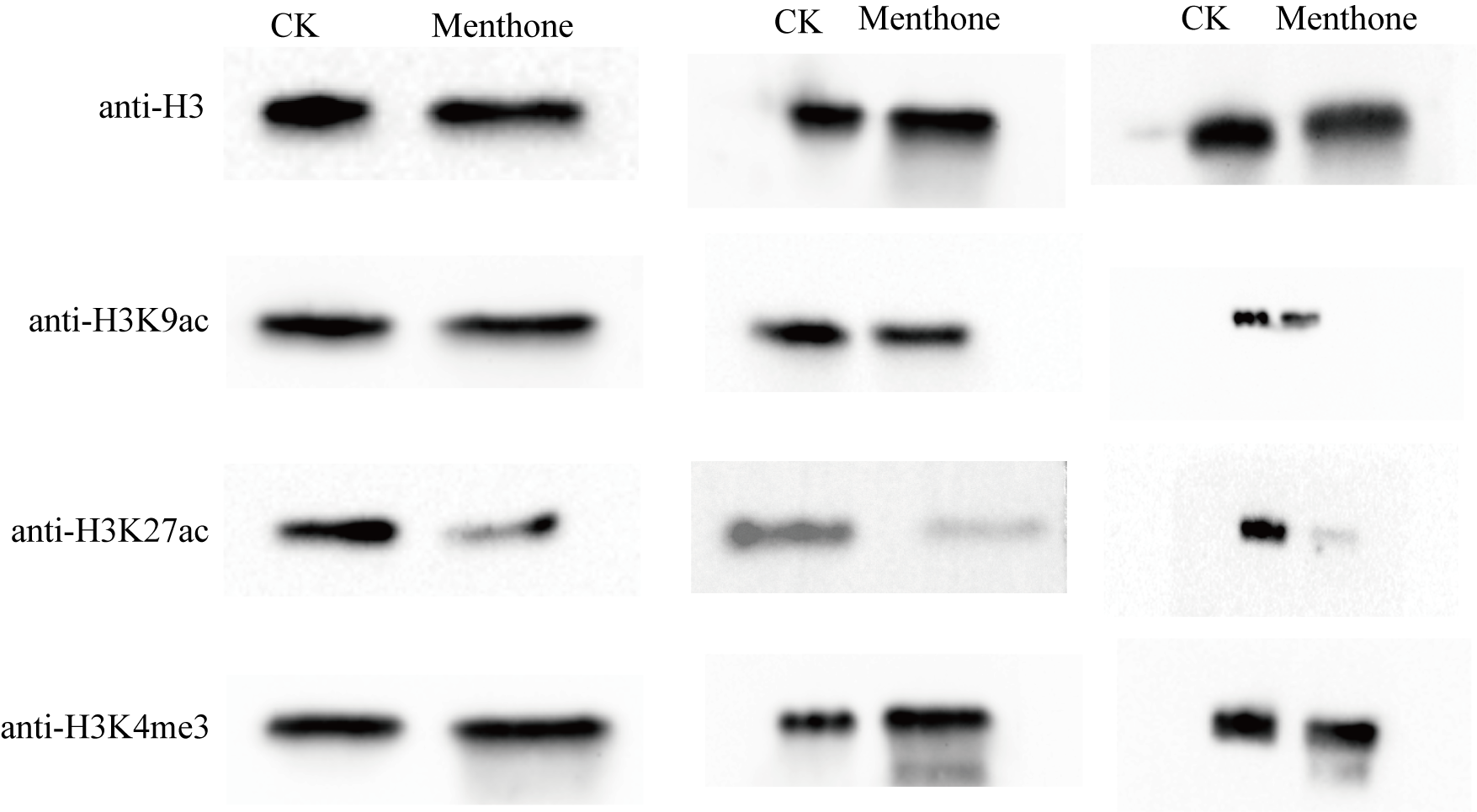


Figure S1 The levels of H3K9ac, H3K27ac, and H3K4me3 in *F. proliferatum* were assessed using the immunoblotting technique. Anti-H3 served as a loading control.


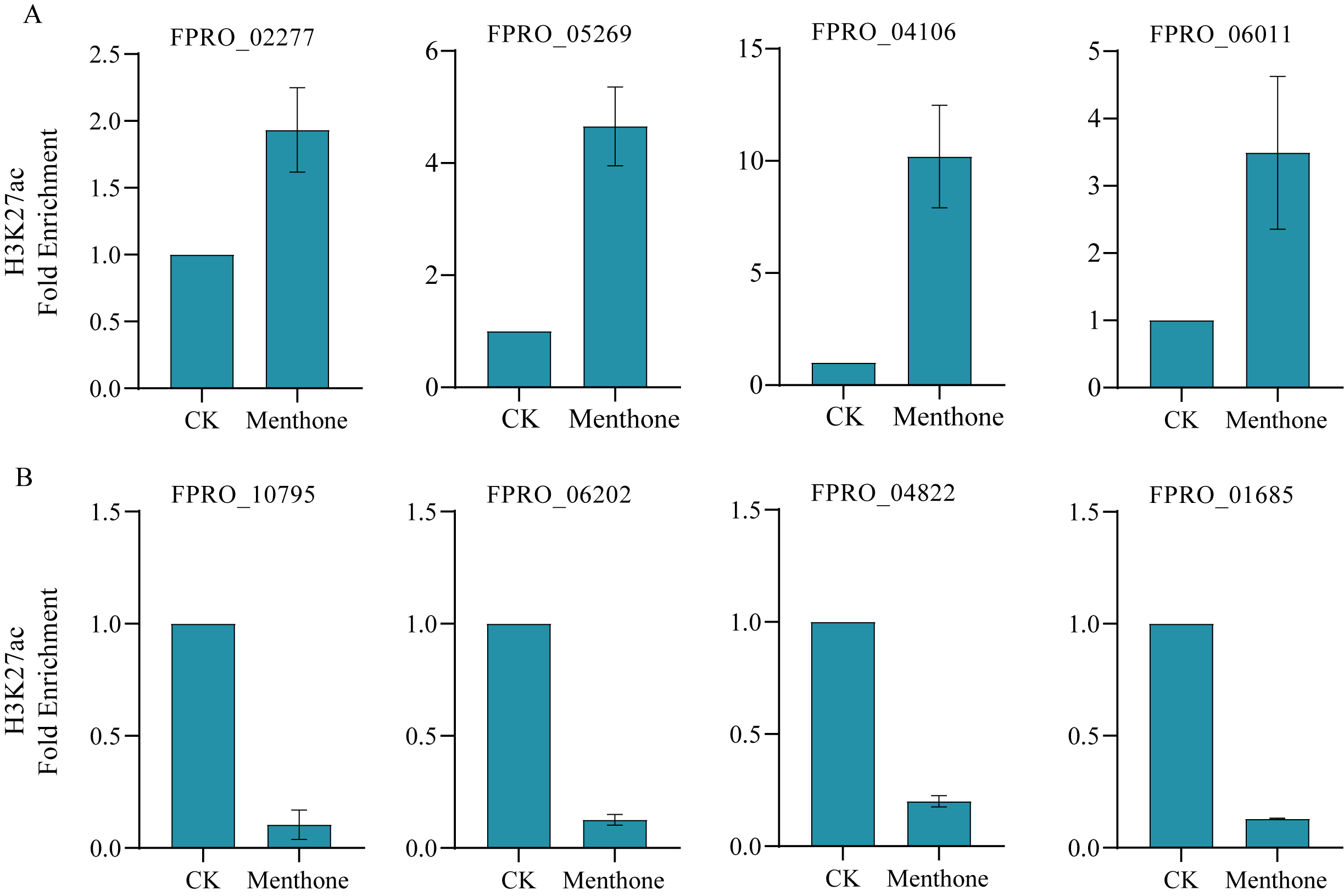


Figure S2 Validation of ChIP-qPCR for H3K27ac-modified enriched peak genes in *F. proliferatum* after menthone treatment. (A) ChIP-qPCR validation of H3K27ac-modified enriched peak genes in *F. proliferatum* following menthone treatment. (B) ChIP-qPCR validation of non-H3K27ac-modified enriched peak genes in *F. proliferatum* after menthone treatment.
